# Determine TB-LAM point-of-care tuberculosis assay predicts poor outcomes in outpatients during their first year of antiretroviral therapy in South Africa

**DOI:** 10.1186/s12879-020-05227-9

**Published:** 2020-07-31

**Authors:** Andrew D. Kerkhoff, Nicky Longley, Nicola Kelly, Anna Cross, Monica Vogt, Robin Wood, Sabine Hermans, Stephen D. Lawn, Thomas S. Harrison

**Affiliations:** 1 Division of HIV, Infectious Diseases and Global Medicine, Zuckerberg San Francisco General Hospital and Trauma Center, University of California, San Francisco, San Francisco, California USA; 2 Desmond Tutu HIV Centre, Institute of Infectious Disease and Molecular Medicine, University of Cape Town, Cape Town, South Africa; 3 Institute of Infection and Immunity, St George’s University of London, London, UK; 4 Department of Clinical Research, Faculty of Infectious and Tropical Diseases, London School of Hygiene and Tropical Medicine, London, UK; 5 Department of Global Health, Academic Medical Centre, Amsterdam Institute for Global Health and Development, University of Amsterdam, Amsterdam, The Netherlands

**Keywords:** Tuberculosis, Lipoarabinomannan, Antiretroviral therapy, Outpatient, Mortality, Africa

## Abstract

**Background:**

Determine TB-LAM is the first point-of-care test (POC) for HIV-associated tuberculosis (TB) and rapidly identifies TB in those at high-risk for short-term mortality. While the relationship between urine-LAM and mortality has been previously described, the outcomes of those undergoing urine-LAM testing have largely been assessed during short follow-up periods within diagnostic accuracy studies. We therefore sought to assess the relationship between baseline urine-LAM results and subsequent hospitalization and mortality under real-world conditions among outpatients in the first year of ART.

**Methods:**

Consecutive, HIV-positive adults with a CD4 count < 100 cells/uL presenting for ART initiation were enrolled. TB diagnoses and outcomes (hospitalization, loss-to-follow and mortality) were recorded during the first year following enrolment. Baseline urine samples were retrospectively tested using the urine-LAM POC assay. Kaplan Meier survival curves were used to assess the cumulative probability of hospitalization or mortality in the first year of follow-up, according to urine-LAM status. Cox regression analyses were performed to determine independent predictors of hospitalization and mortality at  three months and one year of follow-up.

**Results:**

468 patients with a median CD4 count of 59 cells/uL were enrolled. There were 140 patients (29.9%) with newly diagnosed TB in the first year of follow-up of which 79 (56.4%) were microbiologically-confirmed. A total of 18% (*n* = 84) required hospital admission and 12.2% (*n* = 57) died within a year of study entry. 38 out of 468 (8.1%) patients retrospectively tested urine-LAM positive – including 19.0% of those with microbiologically-proven TB diagnoses (*n* = 15/79) and 23.0% (*n* = 14/61) of those with clinical-only TB diagnoses; 9 of 38 (23.7%) of patients retrospectively testing LAM positive were never diagnosed with TB under routine program conditions. Among all patients (*n* = 468) in the first year of follow-up, a positive urine-LAM result was strongly associated with all-cause hospitalization and mortality with a corresponding adjusted hazard ratio (aHR) of 3.7 (95%CI, 1.9–7.1) and 2.6 (95%, 1.2–5.7), respectively.

**Conclusions:**

Systematic urine-LAM testing among ART-naïve HIV-positive outpatients with CD4 counts < 100 cells/uL detected TB cases that were missed under routine programme conditions and was highly predictive for subsequent hospitalization and mortality in the first year of ART.

## Background

Tuberculosis (TB) remains the leading cause of death among people living with HIV (PLHIV) [1], especially during the initial months of antiretroviral therapy (ART), and is a key driver of morbidity and mortality in ART programmes [2]. Both prevalent and incident TB cases are concentrated in patients accessing ART services for the first time and during the initial months of treatment [3–6].

The proportion of patients being diagnosed with TB at enrolment in ART clinics has increased in recent years, in part due to implementation of systematic, symptom-based screening [1]. The World Health Organization (WHO), recommends that all PLHIV be screened at each clinical encounter for the presence of cough, fever, weight loss or night sweats and that those with any one symptom (of any duration) undergo further microbiologic testing with either Xpert MTB/RIF or smear microscopy where Xpert is unavailable [7]. However, PLHIV can often have non-specific presentations and may not manifest typical TB symptoms. This is reflected in the diagnostic performance of the WHO-symptom screening rule; while it approaches 90% sensitivity in ART-naïve patients, it’s specificity among such individuals is only 28% [8]. Symptom based-screening therefore still fails to detect more than 10% of all individuals with HIV-associated TB prior to ART initiation and its poor specificity makes it challenging to determine who to prioritize for further TB investigations, as systematic testing of all PLHIV screening positive in low-resource settings may not be feasible. Delayed and missed TB diagnoses prior to ART initiation are key barriers to reducing HIV-associated TB mortality in ART programmes and thus there is significant interest in developing improved TB screening and testing strategies [9].

The Alere Determine TB-LAM assay (Abbott Diagnostics, Lake Bluff, IL,USA; henceforth ‘urine-LAM’) is a simple lateral-flow, point-of-care test that detects mycobacterial lipoarabinomannan in urine samples [10]. While its overall diagnostic accuracy is limited (pooled sensitivity 42%), it has moderate sensitivity (54%) in those with advanced HIV disease (CD4 counts ≤100 cells/μL) [11]. Notably, several studies have demonstrated the prognostic value of urine-LAM, including a meta-analysis, which found a positive urine-LAM result to be associated with a two-fold increased mortality at  two to three months, even after adjusting for additional covariates, including CD4 cell count [12]. Furthermore, two randomized trials in sub-Saharan Africa found that the use of urine-LAM in addition to routine investigations was associated with a survival benefit in high-risk inpatients with advanced HIV [13, 14]. However, the predominance of studies to-date have evaluated short-term outcomes among patients undergoing urine-LAM testing, with only a few studies assessing the relationship between urine-LAM status and clinical outcomes beyond three months [15–20]. We therefore evaluated the prognostic value of systematic urine-LAM testing among HIV outpatients with CD4 counts < 100 cells/μL who were presenting to ART services for the first time and for whom very complete clinical outcomes up to one year of follow-up were available, including TB diagnoses, hospitalization records, losses-to-follow-up, and death.

## Methods

This was a sub-study from a prospective cohort of ART-naïve adults in whom systematic cryptococcal antigen (CrAg) screening was implemented [21]. For the parent study, all newly diagnosed, consecutive HIV positive patients presenting for ART to 2 outpatient clinics in Cape Town, South Africa between May 2011 and April 2014 patients were eligible if they were ≥ 18 years of age at the time of enrollment, ART-naïve, had a CD4 cell count ≤100 cells/μL and no prior history of Cryptococcal disease. This sub-study was restricted to those who did not have a known TB diagnosis at the time of study entry. All participants had baseline blood and urine samples obtained and had data collected prospectively at study enrollment and every three months for the first year of ART using data collected from patient records, telephone or home visits [21]. Fresh clinical samples were tested for the presence of CrAg as described below and any remaining volumes were stored at -80C until further testing was performed.

Prospective data including routine clinic blood investigations, ART management, hospital admissions, TB diagnosis (clinical, radiological, histological and microbiological), opportunistic infections and outcomes (death, hospitalization and lost-to-follow up) were collected on all patients at three-monthly intervals for their first year of care. As per the local HIV guidelines at the time when the study was undertaken, all patients were started on an efavirenz-based ART regimen within  two to four weeks following their first clinic appointment, unless there was a specific clinical indication to do otherwise [22]. No clinical specimens were routinely obtained during follow-up visits. The study was approved by the Research and Ethics Committee of the University of Cape Town and all patients provided written informed consent in their primary language.

### Laboratory procedures

All patients with a clinical suspicion of TB (fever, night sweats, weight loss, cough  at least two weeks duration) had samples sent to the laboratory by the clinic staff. The specific investigations that were ordered varied from patient to patient and correlated with the local policy at the time that the patient was enrolled in the clinic [22]. The large majority of specimens collected for mycobacteriology were sputum samples, however non-respiratory samples, including cerebrospinal fluid, pleural fluid, blood, urine and lymph node fine needle aspirates were collected as clinically indicated. Investigations available in the laboratory included: Xpert MTB/RIF assay (Cepheid inc., Sunnyvale, CA, USA), smear florescence microscopy and automated liquid culture using mycobacterial growth indicator tubes (MGIT 960, Becton Dickinson, Sparks, Maryland, USA). Frozen urine specimens collected prior to ART initiation were thawed and tested retrospectively in May 2014 for the presence of lipoarabinnomannan (LAM) using the Determine TB-LAM assay per manufacturer instructions; only 17 (3.6%) of samples that were urine CrAg positive had previously undergone a freeze-thaw cycle to determine CrAg titres. Three of the four trained personnel (NK, NL, MV, SDL) read the results for each specimen independently and a majority consensus was reached. A positive LAM result was defined using grade 2 cut-off on the old manufacturer’s card as this was the basis of expert opinion and WHO guidelines at the time [23]; this corresponds with a grade 1 cut-off using the current manufacturer’s card. Baseline serum, plasma, urine, whole blood and CSF (where available) were tested prospectively for CrAg using a lateral flow assay (LFA) (IMMY, Oklahoma, USA) and those screening positive had CrAg titres determined using the CrAg LFA-IMMY kit, as previously described [21].

### TB-related definitions

‘Newly diagnosed TB’ was defined as any patient with a new TB diagnosis at any point between study entry and up to one year after enrolment, independent of whether the diagnosis was based on microbiological, clinical or radiological evidence. The term ‘incident TB’ was intentionally not utilized, as patients were not systematically tested for active TB at study entry and thus new diagnoses during ART could reflect either prevalent TB disease that was missed at study entry or incident disease that developed while taking ART. A ‘microbiological TB diagnosis’ was defined by the detection of *M. tuberculosis* by Xpert MTB/RIF or culture on any clinical sample; notably, the detection of acid-fast bacilli (AFB) by sputum microscopy was not considered a microbiologically-confirmed TB case unless TB was also detected by culture or Xpert. A “clinical TB diagnosis” was defined as any patient who was started on TB therapy without the presence of a positive Xpert or culture result.

### Outcomes of interest

In addition to data gathered during follow up visits, dates of TB diagnoses and TB therapy (if applicable) as well as clinical outcomes (hospitalization, mortality) were also collected from the National Health Laboratory Service (NHLS) electronic results database, the Department of Home Affairs Death Register and the provincial Government electronic database systems (eKapa and Tier.net). All patients who missed one of their three-monthly clinic visits were traced by study staff using telephone calls and home visits. Any patient whom could not be confirmed as either alive or dead at one year of follow-up using any of the above sources of information was defined as ‘LTFU.'

A ‘composite adverse outcome,’ was defined as any patient who required hospitalization for any reason (all-cause), was LTFU and/or died (all-cause) within one year of ART initiation (or one year after study entry for the small proportion who did not start ART). For the purposes of evaluating the relationship between urine-LAM status and outcomes, we categorized patients into one of three mutually-exclusive groups: urine-LAM positive, urine-LAM negative/TB diagnosed in the first year of follow-up, or urine-LAM negative/no TB diagnosed in the first year of follow-up.

### Statistical analysis

Data were analysed using Stata version 14.0 (StataCorp, College Station, Texas, USA). Proportions and medians were compared using either Pearson’s chi-squared, Fisher’s exact and Wilcoxon rank-sum tests as appropriate. Cox regression analysis was performed to evaluate the association between urine-LAM positivity and hospitalization as well as mortality at three months and one year of follow-up. Person-time was accrued from the date of study enrolment until death, lost-to-follow-up, first hospitalization (in hospitalization-related analyses) or censorship 365 days after study enrolment. Directed acyclic graphs (DAG) were used to identify confounders of the relationship between urine-LAM and hospitalization and urine-LAM and mortality [24]; therefore, a priori covariates for both hospitalization and mortality were age, hemoglobin level, and CD4 count based on the minimal sufficient adjustment sets. It is not known whether sex may be causally associated with urine-LAM status; given such uncertainty, a change in estimate approach was utilized and sex was only included if the adjusted urine-LAM point estimate differed by > 10%, so as to not reduce statistical precision. Schoenfeld’s global test was used to test the proportional hazards assumption. Kaplan Meier survival curves were made to visualize the cumulative incidence and timing of TB treatment initiation as well as the cumulative incidence of adverse clinical outcomes (hospitalization or mortality) in the first year of follow-up, according to urine-LAM status.

## Results

### Patient characteristics

**Fig. 1:**
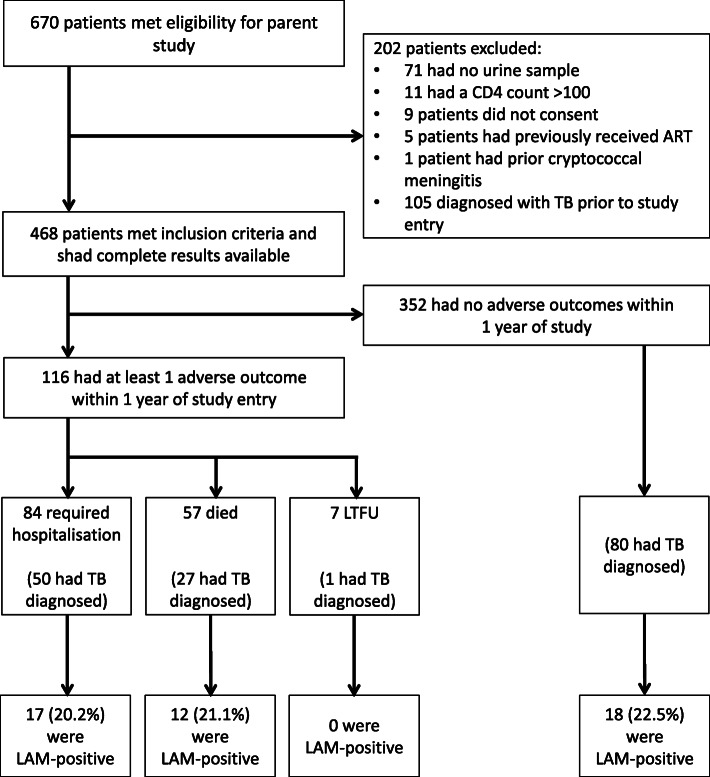
Flow diagram showing the study population, the number of patients included as well as urine-LAM results, adverse outcomes, and TB diagnoses within  one year of ART initiation. Hospitalizations and deaths were not mutually exclusive, such that some patients were hospitalized and subsequently died. Therefore, the total number of patients with newly diagnosed TB was 140 and the total number of patients with a positive urine-LAM result was 38

Six hundred seventy patients were identified for possible inclusion in the parent study of whom 468 met inclusion criteria and had complete results available for this sub-study (Fig. 1). Among the 202 patients excluded, 105 had a known TB diagnosis and were receiving TB treatment at the time of study entry, of which 41 (40.0%) retrospectively tested urine-LAM positive.

Patients included in the study (*n* = 468) were young (median 35 years old) and the majority were women (55%). They had advanced immunosuppression (median CD4 59 cells/μL) and tended to be anemic (Table 1). More than 90% of all patients started ART, with a median time to ART initiation of 15 days (IQR, 11–26). Baseline characteristics did not differ between the two study sites and thus data from two sites were combined for all subsequent analyses (data not shown).

**Table 1:** Baseline characteristics among patients not on tuberculosis therapy at study entry (*n* = 468)

	All patients (*n* = 468)
**Age, median (IQR**)	35.4 (30.2–40.8)
**Sex**	
Male	209 (44.7)
Female	259 (55.3)
**CD4 cell count (cells/μL)**	
Median (IQR)	59 (32–80)
< 50	193 (41.2)
50–99	275 (58.8)
**Hemoglobin (g/dL)**^a^	
Median (IQR)	11.0 (9.3–12.5)
Anemia severity	
Moderate/severe	209 (47.2)
None/mild	234 (52.8)
**Creatinine (μmol/L)**^b^	68 (58–83)

*Abbreviations*: *IQR* interquartile range

^a^n = 443; ^b^n = 425

### Overall TB diagnoses

There were 140 patients with a TB diagnosis made (either microbiological or clinical) within the first year of study entry for a combined prevalence and incidence of 29.9% (95%, 26.0–34.3); however, only 79 (56.4%) of such patients had a microbiologically-proven diagnosis by either Xpert or culture.

### TB diagnosis characteristics stratified according to urine-LAM assay result

Overall, 38/468 (8.1%) patients retrospectively tested urine-LAM positive (Table 2), of which three-quarters (76.3%; *n* = 29/38) were prospectively diagnosed with TB (either microbiological or clinical) within one year of enrolment. Among those with microbiologically-proven TB (*n* = 79) and a clinical-only TB diagnosis (*n* = 61), urine-LAM retrospectively detected TB in 15 (19.0%) and 14 (23.0%) patients, respectively. Urine-LAM positive patients were less likely to have microbiologically-proven TB compared to patients diagnosed with TB, testing urine-LAM negative (39.5% vs. 57.7%; *p* = 0.05) (Table 2). Notably, patients who retrospectively tested urine-LAM positive were substantially less likely to have had a clinical specimen undergo Xpert or culture-based testing for TB in the first year of follow-up (47.4% vs. 81.1%, *p* < 0.001, supplementary Table 1).

**Table 2:** Diagnosis and treatment outcomes by baseline LAM status among patients not on anti-TB treatment at study entry (*n* = 468)

	All patients (*n* = 468)	LAM positive (*n* = 38)	LAM Negative/TB diagnosis (*n* = 111)	LAM Negative/No TB diagnosis (*n* = 319)	*P*-value*
**Tuberculosis diagnoses within one year of ART**					
TB diagnosis (microbiological or clinical)					
Yes	140 (29.9)	29 (76.3)	111 (100)	0	< 0.001
No	328 (70.1)	9 (23.7)	0	319 (100)	
Time to TB diagnosis (median [IQR] in days; microbiological or clinical)^a^	14 (2–36)	5 (0–13)	16 (3–45)	–	0.005
TB diagnosis (microbiologically-proven only)					
Yes	79 (16.9)	15 (39.5)	64 (57.7)	0	0.053
No	389 (83.1)	23 (60.5)	47 (42.3)	319 (100)	
Time to TB diagnosis (median [IQR] in days; microbiologically-proven only)^b^	15 (3–36)	5 (2–13)	21 (3–41)	–	0.025
TB diagnosis (clinical only)					
Yes	61 (13.0)	14 (36.8)	47 (42.3)	0	0.55
No	407 (87.0)	24 (63.2)	64 (57.7)	319 (100)	
Time to TB diagnosis (median [IQR] in days; clinical only)^c^	14 (0–36)	4 (0–18)	15 (1–70)	–	0.10
**TB and HIV treatment**					
Started TB treatment (within one year of study entry among all patients)					
Yes^#^	134 (28.6)	25 (65.8)	109 (98.2)	0	< 0.001
No	334 (71.6)	13 (34.2)	2 (1.8)	319 (100)	
Time to initiation of TB treatment (median [IQR] in days)^d^	17 (5–42)	7 (1–14)	23 (7–51)	–	0.001
TB treatment outcomes (among those starting within one year of study entry)^d^					
Completed	94 (70.7)	16 (64.0)	78 (72.2)	–	0.53
Did not complete	9 (6.8)	1 (4.0)	8 (7.4)	–	
Died on therapy	19 (14.3)	6 (24.0)	13 (12.0)		
Unknown	11 (8.3)	2 (8.0)	9 (8.3)	–	
Started ART (within one year of study entry)^e^					
Yes	430 (91.9)	27 (71.1)	105 (94.6)	298 (93.4)	< 0.001
No	38 (8.1)	11 (29.0)	6 (5.4)	21 (6.6)	
Time to initiation of ART (median [IQR] in days)^e^	15 (11–25)	23 (14–32)	21 (14–35)	14 (9–22)	< 0.001
ART status within one year of study entry (among those who started)^e^					
On ART, no switch, did not default	344 (80.0)	22 (81.5)	85 (81.0)	237 (79.5)	0.95
On ART, switched	32 (7.4)	3 (11.1)	8 (7.6)	21 (7.1)	
Off ART, defaulted	50 (11.6)	2 (7.4)	11 (10.5)	37 (12.4)	
Unknown	4 (0.9)	0	1 (1.0)	3 (1.0)	

*In analyses restricted to among patients with microbiologically-confirmed TB (n = 79), *p*-values reference the comparison between LAM positive patients and LAM negative, TB-positive patients only

*Abbreviations*: *ART* antiretroviral therapy, *TB* tuberculosis

^a^n = 140; ^b^*n* = 79;^c^n = 61;^d^n = 133^e^n = 430

^#^6 patients with a TB diagnosis within one year of study entry were not known to have started TB therapy, 3 patients had unknown TB treatment status (1 LAM positive), 3 patients died prior to starting TB therapy (3 LAM positive)

Of patients prospectively diagnosed with TB in the first year of ART (*n* = 140), those who tested urine-LAM positive had earlier TB diagnoses compared those who tested urine-LAM negative (5 days vs. 16 days, *p* = 0.005); the timing of TB diagnoses did not differ substantially between those who had a microbiological diagnosis vs. a clinical-only diagnosis (Table 2). Among urine-LAM positive patients who were prospectively diagnosed with TB, 7 (24.1%) were diagnosed more than 14 days after study entry (maximum 186 days).

9 of 38 (23.7%) patients found to be urine-LAM positive at study entry did not have a TB diagnosis (clinical or microbiological) made under routine program conditions at any time point in the first year following study enrolment (supplementary Table 2). Their median CD4 count was 75 cells/μL (IQR, 24–80) and median hemoglobin concentration was 10.5 g/dL (IQR, 8.0–12.7). Five of the 9 patients were LAM grade 3 positive or greater, strongly suggesting a true positive result, including one of whom died less than 10 days after study enrolment. An additional patient died within two weeks of enrolment after subsequently presenting to the hospital with sepsis physiology. The remaining 3 of 9 urine-LAM positive patients without a TB diagnosis were never hospitalized and remained alive at one year after study entry without known receipt of anti-TB therapy, suggesting the possibility of a false positive result, although it should be considered that these patients did experience immune reconstitution in the setting of ART initiation. No patients were diagnosed with non-tuberculous Mycobacterial infection, Cryptococcus, or other opportunistic infections; while patients were systematically screened for the presence of Cryptococcal infection at study entry, they were not systematically investigated for non-tuberculous mycobacteria or other opportunistic infections.

### TB treatment characteristics stratified according to urine-LAM assay result

Among the 140 patients with a new TB diagnosis in the first year of follow-up, 134 (95.7%) were subsequently started on anti-TB therapy (Table 2). Notably, less than two-thirds of patients retrospectively testing urine-LAM positive were started on TB therapy compared to nearly all (98.2%) of those with a urine-LAM negative TB diagnosis (Table 2). In addition to the 9 patients who were not diagnosed with TB in the first year of follow-up (and therefore were not started on TB treatment), there were 4 patients who were urine-LAM positive and diagnosed with TB, but were not known to have started TB treatment (supplementary Table 2). Three of 4 patients died prior to receiving TB treatment, while the fourth was not known to have started TB treatment but was alive at one year of follow-up. Among patients who were started on TB treatment (*n* = 134), the proportion who completed TB therapy did not differ by urine-LAM result (64.0% vs. 72.2%, *p* = 0.53; Table 2). Patients testing urine-LAM positive were also less likely to have been initiated on ART compared to those testing urine-LAM negative (71.1% vs. 94.6%, *p* < 0.002; Table 2); however, among those who started ART, ART-status at one year did not differ according to the urine-LAM result (Table 2).

**Fig. 2:**
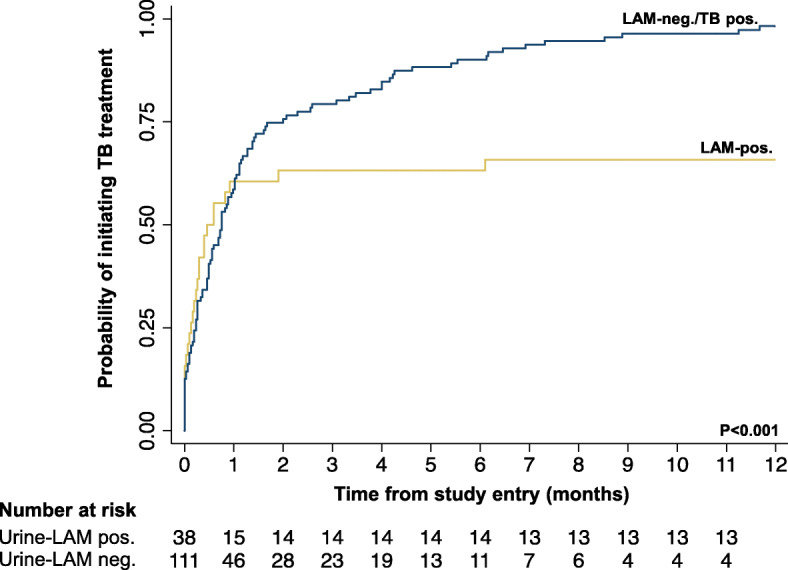
Cumulative probability of starting tuberculosis treatment within one year of study entry according to urine-LAM result (*n* = 149)

Of patients starting TB treatment (n = 134), the median time to initiation of treatment was 17 days (IQR, 5–42). While patients who retrospectively tested urine-LAM positive were substantially less likely to have been started on TB treatment, those starting TB treatment were more rapidly initiated on treatment compared to patients who tested urine-LAM negative (7 days vs. 23 days, *p* < 0.001; Table 2; Fig. 2).

### Overall outcomes

Overall, 116 (24.8%) of patients had at least one adverse outcome within one year of study entry (‘composite adverse outcome’). This comprised 84 (17.3%) persons who required hospitalization, 57 (12.2%) who died, and 7 (1.5%) who were LTFU (Fig. 1). Among all patients, the median time to hospitalization was 39 days (IQR, 13–129), while the median time to death was 74 days (IQR, 25–140).

### Relationship between urine-LAM result and all-cause hospitalization in the first year of follow-up

**Fig. 3:**
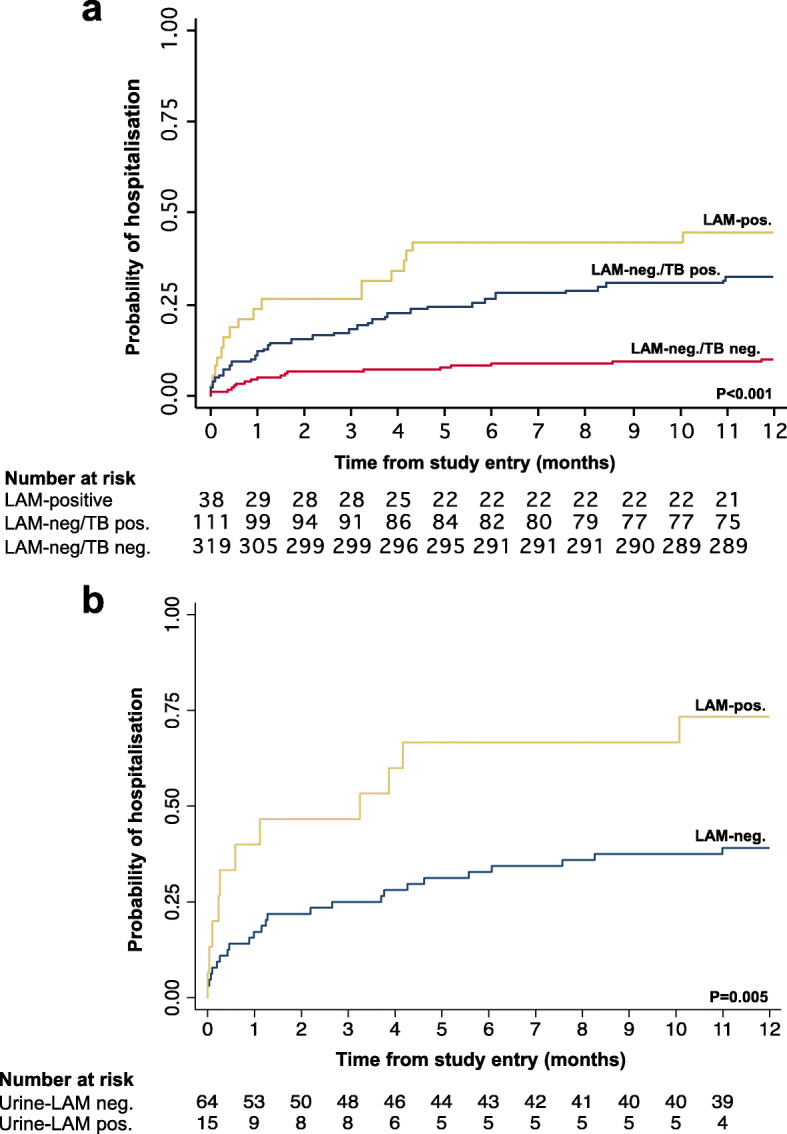
Cumulative probability of hospital admission within one year of study entry according to urine-LAM and tuberculosis diagnosis category among **a**) all patients (*n* = 468), **b**) patients with with new microbiological TB diagnosis in the first year of study entry (*n* = 79)

We evaluated the relationship between urine-LAM result and TB diagnosis (either microbiological or clinical) in the first year of enrolment with the patient’s clinical outcomes (Table 3). Overall, urine-LAM positive patients had poorer outcomes and tended to experience them sooner after study entry, compared to those who tested urine-LAM negative (either with or without a TB diagnosis) (Table 3). Nearly one-third and one-half of patients retrospectively testing urine-LAM positive required hospitalization within  three months and one year following study entry, respectively; this was a significantly larger proportion than either urine-LAM negative/TB-positive patients or urine-LAM negative/ TB-negative patients (Table 3; Fig. 3a). Urine-LAM positive patients accounted for more than 20% (*n* = 17/84) of all-cause hospitalization within the first year of study enrolment in this cohort (Table 3).

**Table 3:** Outcomes by baseline urine-LAM status among patients not on anti-TB treatment at study entry (*n* = 468)

	All patients (*n* = 468)	LAM positive (*n* = 38)	LAM Negative/TB diagnosed (*n* = 111)	LAM Negative/No TB diagnosed (*n* = 319)	*p*-value
**Clinical outcome at three months**					
Required hospitalization	50 (10.7)	10 (26.3)	20 (18.0)	20 (6.3)	< 0.001
Hospital readmission	4 (0.9)	0	2 (1.8)	2 (0.6)	0.48
Vital status					
Dead	31 (6.6)	11 (29.0)	6 (5.4)	14 (4.4)	< 0.001
LTFU	2 (0.4)	0	0	2 (0.6)	
Alive	435 (93.0)	27 (71.1)	105 (94.6)	303 (95.0)	
**Clinical outcome at six months**					
Required hospitalization	73 (15.6)	16 (42.1)	29 (26.1)	28 (8.8)	< 0.001
Hospital readmission	6 (1.3)	1 (2.6)	3 (2.7)	2 (0.6)	0.14
Vital status					
Dead	44 (9.4)	12 (31.6)	11 (9.9)	21 (6.6)	< 0.001
LTFU	2 (0.4)	0	0	2 (0.6)	
Alive	422 (90.2)	26 (68.4)	100 (90.1)	296 (92.8)	
**Clinical outcome at one year**					
Required hospitalization	84 (18.0)	17 (44.7)	36 (32.4)	31 (9.7)	< 0.001
Median time to hospitalization (IQR), (median [IQR] in days)^a^	39 (13–129)	18 (4–118)	74 (14–166)	31 (14–149)	0.39
Hospital readmission	10 (2.1)	1 (2.6)	5 (4.5)	4 (1.3)	0.10
Vital status					
Dead	57 (12.2)	12 (31.6)	17 (15.3)	28 (8.8)	0.002
LTFU	7 (1.5)	0	1 (0.9)	6 (1.9)	
Alive	404 (86.3)	26 (68.4)	93 (83.8)	285 (89.3)	
Median time to death (IQR), (median [IQR] in days)^b^	74 (25–140)	19 (8–65)	124 (38–259)	90 (27–160)	0.002
Died without starting TB treatment (among all patients)	35 (7.5)	5 (13.2)	2 (1.8)	28 (8.8)	0.021

*Abbreviations*: *LTFU* lost-to-follow-up

^a^n = 84; ^b^n = 57

A positive urine-LAM result was strongly associated with 90-day (adjusted hazard ratio = [aHR] = 3.1) and one year all-cause hospitalization (aHR = 3.7) (Table 4); TB patients who tested urine-LAM negative had lower hospitalization rates compared to urine-LAM positive patients (aHR = 2.0 and 2.4 at 90 days and one year, respectively), but such patients were still more likely to be hospitalized than patients without a TB diagnosis (Table 4). When the analysis was restricted to among those with a microbiologically-confirmed diagnosis of TB (*n* = 79), a positive urine-LAM result was associated with a higher cumulative incidence of hospitalization in the first year of follow-up compared to those testing urine-LAM negative (Fig. 3b); in such patients a positive urine-LAM result did not, however, independently predict need for subsequent hospitalization (Table 4).

**Table 4:** Cox regression analyses for hospitalization and mortality in the first year of ART according to urine-LAM result and TB diagnosis category among ART-naïve adults

	Unadjusted HR (95% CI)	*p*-value	Adjusted HR (95% CI)	*P*-value
**Hospitalization at 90 days**				
No TB diagnosed	1.0	< 0.001	1.0	0.025
Urine-LAM negative/TB diagnosed	2.88 (1.55–5.35)		1.97 (1.02–3.81)	
Urine-LAM positive	5.36 (2.51–11.46)		3.09 (1.33–7.17)	
**Hospitalization at one year**				
No TB diagnosed	1.0	< 0.001	1.0	< 0.001
Urine-LAM negative/TB diagnosed	3.35 (2.07–5.41)		2.42 (1.45–4.06)	
Urine-LAM positive	5.95 (3.29–10.58)		3.65 (1.89–7.06)	
**Hospitalization at 90 days among those with microbiologically-confirmed TB (n = 79)**				
Urine-LAM negative	1.0	0.07	1.0	0.29
Urine-LAM positive	2.44 (1.00–5.93)		1.75 (0.64–4.78)	
**Hospitalization at one year among those with microbiologically-confirmed TB (*n*** **= 79)**				
Urine-LAM negative	1.0	0.019	1.0	0.16
Urine-LAM positive	2.49 (1.22–5.06)		1.84 (0.80–4.27)	
**Mortality at 90 days**				
No TB diagnosed	1.0	< 0.001	1.0	0.015
Urine-LAM negative/TB diagnosed	1.23 (0.47–3.20)		1.01 (0.37–2.74)	
Urine-LAM positive	7.67 (3.48–16.90)		3.83 (1.52–9.66)	
**Mortality at one year**				
No TB diagnosed	1.0		1.0	0.07
Urine-LAM negative/TB diagnosed	1		1.40 (0.74–2.67)	
Urine-LAM positive			2.60 (1.19–5.66)	
**Mortality at 90 days among those with microbiologically-confirmed TB (n = 79)**				
Urine-LAM negative	1.0	0.016	1.0	0.07
Urine-LAM positive	4.95 (1.43–17.12)		3.90 (0.91–16.72)	
**Mortality at one year among those with microbiologically-confirmed TB (n = 79)**				
Urine-LAM negative	1.0	0.035	1.0	0.10
Urine-LAM positive	3.23 (1.17–8.90)		2.84 (0.86–9.41)	

All analyses among n = 468 patients unless explicitly stated otherwise

*P*-value based on likelihood-ratio test

### Relationship between urine-LAM result and all-cause mortality in the first year of follow-up

**Fig. 4:**
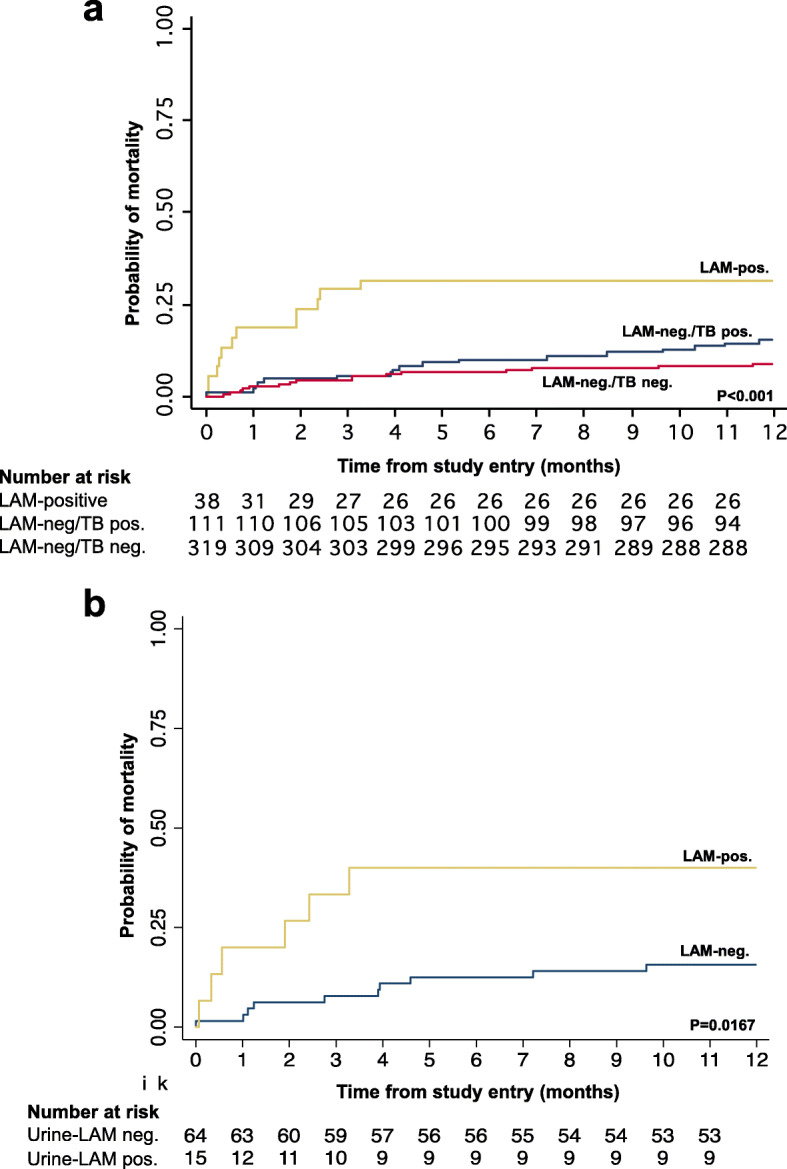
Cumulative probability of mortality according to urine-LAM and tuberculosis diagnosis category among **a)** all patients (n = 468), **b)** patients with new microbiological TB diagnosis in the first year of study entry (n = 79)

Nearly one-third (*n* = 12, 31.6%) of patients retrospectively testing urine-LAM positive died within a year of enrolment; this was compared to 15.3% (n = 17) of patients who were urine-LAM negative, but had TB diagnosis within a year of study enrolment and 8.8% (*n* = 28) among those without a TB diagnosis in the first year of follow-up (*p* = 0.002; Table 3; Fig. 4a). Patients testing urine-LAM positive accounted for 36% (*n* = 11/31) of all deaths occurring within 90 days and 21% (n = 12/57) of all deaths occurring during the first year. Notably, 5 of 13 (38.5%) urine-LAM positive patients who were never started on TB treatment died in the first year of follow-up, of which 4 died more than a week after study entry (supplementary Table 2). There were 25 urine-LAM positive patients who were prospectively diagnosed with TB and started on TB treatment; of the 6 patients started on TB treatment within 72 h of study entry, only 1 died (16.7%), compared to 6 (31.6%) of 19 patients who died after being started on TB treatment more than 72 h after study entry.

In multivariable analyses, a positive urine-LAM result was strongly associated with an increased hazard of mortality at 90 days (aHR = 3.8) and one year (aHR = 2.6; Table 4). Among those with microbiologically-confirmed TB (*n* = 79), a positive urine-LAM result was associated with a higher cumulative mortality incidence compared to those with a negative urine-LAM result (Fig. 4b) and there was a trend towards an increased hazard of death at 90 days and one year (Table 4).

## Discussion

This study found that among ambulatory, HIV positive outpatients with severe baseline immunosuppression (CD4 < 100 cells/μL) in the first year of ART, nearly one-third had a new TB diagnosis (prevalent or incident TB) and one-quarter had at least one adverse outcome, including need for hospitalization, LTFU, or death. A positive urine-LAM result prior to ART initiation (tested retrospectively) was strongly and independently associated with the need for hospitalization as well as mortality during the first year of follow-up. These results add to prior studies demonstrating the prognostic value of urine-LAM testing among HIV-positive outpatients with advanced immunodeficiency [16–20, 25].

This was a pragmatic study undertaken in real-world clinical conditions without systematic TB testing at baseline and allowed us to determine that approximately one-quarter of patients who retrospectively tested LAM positive were never diagnosed with TB and more than one-third were never started on anti-TB therapy. Nearly half of all patients testing urine-LAM positive ultimately required hospitalization in the first year of ART. Furthermore, 12 patients testing urine-LAM positive prior to ART initiation died within a year of enrolment; 5 died without being started on TB treatment (4 of which died more than one week after study entry), while an additional 6 died among those who had delayed initiation of TB treatment (> 72 h after study entry). Ultimately, patients testing urine-LAM positive accounted for 35% and 21% of all-cause deaths occurring within 90 days and one year of enrolment, respectively, despite accounting for just 8% of the overall study population.

Previous studies among both inpatients and outpatients have shown urine-LAM to be an independent predictor of poor clinical characteristics, including early mortality [12, 26]. In the present study, we found that a positive urine-LAM result prior to ART initiation was a strong predictor of the need for hospitalization as well as subsequent mortality in the first year following ART initiation. This is consistent with prior observational outpatient studies that have also found that a positive urine-LAM result predicts six-month and one-year mortality during ART [16–20, 25]. However, in each of these studies, including the present study, mortality in those who tested urine-LAM positive was largely restricted to within three months of ART initiation. The association between positive urine-LAM testing and an increased risk of mortality in these studies likely reflects high mycobacterial burden and disseminated TB in the context of advanced HIV-associated immunodeficiency [27, 28]. While many urine-LAM positive patients may have direct renal involvement with TB, especially those with advanced HIV [29], there is increasing evidence that LAM antigeneuria might also reflect glomerular filtration of systemically circulating LAM (or LAM components) [30, 31].

Based on an updated appraisal of the literature [11], WHO guidelines have been recently revised to now recommend urine-LAM testing be considered among PLHIV presenting to outpatient settings with 1) signs and symptoms of TB and/or who are seriously ill, and 2) in those who have CD4 counts < 100 cells/μL, irrespective of TB signs and symptoms [32]. Despite initial WHO recommendations for urine-LAM testing for the evaluation of HIV-associated TB being made in 2015, only a small number of high-burden countries have incorporated urine-LAM testing into the TB diagnostic algorithm [33, 34]. Even where urine-LAM testing has been implemented, it is rarely utilized among outpatients. Diagnostic studies among ART-naïve outpatients have previously found that urine-LAM’s use increased diagnostic yield [20, 35, 36]. However, when urine-LAM was evaluated among more immunocompetent ambulatory patients, including those who are ART-experienced [18] and in the setting of community case finding studies [37], its additive yield was marginal.

While urine-LAM may increase overall TB diagnostic yield, its greatest clinical utility lies in its ability to rapidly detect TB in less than 30 min in PLHIV who are at greatest risk for morbidity and mortality [33]. Among outpatients, urine-LAM is likely to have greatest impact in settings where same-day evaluations and diagnostics (chest X-ray, sputum microscopy and sputum Xpert) are not available, as its use would allow for immediate same-day initiation of potentially lifesaving therapy [20]; however, the TB FAST-TRACK study found that urine-LAM testing as part of a point-of-care TB algorithm to enable nurses at primary health-care clinics to initiate empirical TB was associated with increased TB treatment coverage, but did not reduce mortality [38]. The reasons underpinning a lack of mortality benefit associated are not fully clear, but TB FAST-TRACK investigators concluded that the development and implementation of more sensitive, point-of-care TB tests that could be feasibly utilized at primary health-care clinics should be prioritized.

Strengths of this study include that patients were consecutively recruited from two different clinic sites, data were prospectively collected up to one year after ART initiation and the study took place under real-world programmatic conditions. Additionally, clinical outcomes at one year were very complete (LTFU was approximately 1.5%) as a result of ascertainment through phone and in-person tracing by study staff as well through extraction from multiple electronic databases. There were however, some weaknesses. The WHO symptom screen was not systematically recorded at baseline as the WHO recommendation (and subsequent roll-out) followed the study’s design. Routine microbiological testing for TB using either Xpert and/or culture was not undertaken as in our previous studies, which did not allow for a reference standard to determine the diagnostic accuracy of the urine-LAM assay or calculation of the true TB prevalence in this patient population. Additionally, because systematic TB testing was not undertaken, 9 urine-LAM positive patients were without a microbiological TB diagnosis and we cannot exclude the possibility of a false positive result in some of these patients. We overall believe that only a small proportion (3 or less) may have represented a false positive result, given that the majority had a strongly positive urine-LAM result, coupled with data from our previous studies in this setting that have demonstrated among both ambulatory and hospitalized patients that the specificity of LAM exceeds 98% when a robust reference standard is utilized [39, 40]. Finally, the apparent yield of urine-LAM in this cohort was lower than might be expected among patients with advanced immunodeficiency given prior evaluations [11]. We believe that this reflect a number of factors, including: 1) a large proportion of patients diagnosed with TB prior to referral for ART initiation and therefore not included in the study - potentially underestimating urine-LAM’s utility in this setting (as evidenced by the finding that approximately 40% of patients excluded due to a known TB diagnosis at study entry were urine-LAM positive), 2) a lack of systematic microbiological investigations for TB prior to ART initiation (including a lower frequency of culture and Xpert testing among urine-LAM positive patients), and 3) systematic urine-LAM testing undertaken among all patients regardless of symptoms (likely lowering the pre-test probability and overall TB prevalence).

## Conclusion

Systematic testing for HIV-associated TB using the urine-LAM point-of-care assay prior to ART initiation was highly predictive for hospitalization and mortality in the first year of ART. Urine-LAM testing among ambulatory adults with CD4 cell counts < 100 cells/μL may help to avert preventable TB-related mortality.

## Supplementary information

**Additional file 1: Supplementary Table 1.** Overview of tuberculosis investigations in the first year of follow-up according to urine-LAM status. **Supplementary Table 2.** Overview of patients testing urine-LAM positive who were not started on tuberculosis therapy in the first year of ART (n=13).

## Data Availability

Data used for this study is available upon request by contacting the corresponding author directly.

## Abbreviations

aHR: Adjusted hazard ratio
ART: Antiretroviral therapy
HIV: Human immunodeficiency virus
IQR: Interquartile range
PLHIV: People living with human immunodeficiency virus
LAM: Lipoarabinomannan
LTFU: Lost-to-follow-up
POC: Point-of-care
TB: Tuberculosis
WHO: World Health Organization
