# Supplementary material for: Determine TB-LAM point-of-care tuberculosis assay predicts poor outcomes in outpatients during their first year of antiretroviral therapy in South Africa
Source: BMC Infect Dis. 2020 Jul 31;20:555. doi: 10.1186/s12879-020-05227-9 (PMC7393716; doi:10.1186/s12879-020-05227-9)
Supplement: Supplementary file 1 — Additional file 1: Supplementary Table 1. Overview of tuberculosis investigations in the first year of follow-up according to urine-LAM status. Supplementary Table 2. Overview of patients testing urine-LAM positive who were not started on tuberculosis therapy in the first year of ART (n=13). [file 12879_2020_5227_MOESM1_ESM.docx]

**Supplementary Table 1.** Overview of tuberculosis investigations in the first year of follow-up according to urine-LAM-status.

|  | **All patients**  **(n=149)** | **LAM positive**  **(n=38)** | **LAM Negative/ TB diagnosis**  **(n=111)** | **p-value** |
| --- | --- | --- | --- | --- |
| **Chest X-ray** |  |  |  |  |
| Suggestive of TB | 54 (36.2) | 12 (31.6) | 42 (37.8) | 0.35 |
| Not suggestive of TB | 4 (2.7) | 0 | 4 (3.6) |  |
| Not undertaken | 91 (61.1) | 26 (68.4) | 65 (58.6) |  |
| **Sputum AFB microscopy** |  |  |  |  |
| Positive | 34 (22.8) | 8 (21.1) | 26 (23.4) | 0.017 |
| Negative | 56 (37.6) | 8 (21.1) | 48 (43.2) |  |
| Not done | 59 (39.6) | 22 (57.9) | 37 (33.3) |  |
| **Xpert on sputum** |  |  |  |  |
| Positive | 55 (36.9) | 11 (29.0) | 44 (39.6) | 0.05 |
| Negative | 20 (13.4) | 2 (5.3) | 18 (16.2) |  |
| Not done | 74 (49.7) | 25 (65.8) | 49 (44.1) |  |
| **Culture on sputum** |  |  |  |  |
| Positive | 46 (30.9) | 10 (26.3) | 36 (32.4) | 0.083 |
| Negative | 26 (17.5) | 3 (7.9) | 23 (20.7) |  |
| Not done | 77 (51.7) | 25 (65.8) | 52 (46.9) |  |
| **Xpert or culture on extra-pulmonary sample*** |  |  |  |  |
| Positive**^#^** | 19 (12.8) | 5 (13.2) | 14 (12.6) | 0.59 |
| Negative | 5 (3.4) | 0 | 5 (4.5) |  |
| Not done | 125 (83.9) | 33 (86.8) | 92 (82.9) |  |
| **Any evidence of rifampicin resistance on any clinical specimen** | 8 (5.4) | 1 (2.6) | 7 (6.3) | 0.68 |

*Xpert or culture performed on any clinical specimen

^#^Among 5 LAM positive patients with positive extra-pulmonary TB investigations, 3 had evidence of Mycobacteraemia,

1 had pleural TB and 1 had TB meningitis. Among 14 TB patients with negative LAM testing, 5 had pleural TB, 4 had TB

lymphadenitis, 3 had Mycobacteraemia, 2 had renal TB.

**Supplementary Table 2. Overview of patients testing urine-LAM positive who were not started on TB therapy in the first year of ART (n=13)**

| **Patient** | **Sex** | **CD4 count**  **(cells/uL)** | **Haemoglobin level (g/dL)** | **LAM Grade** | **Chest X-ray** | **Smear microscopy** | **Underwent microbiological testing** | **TB diagnosis, timing?*** | **TB Treatment started?** | **Death, timing*** |
| --- | --- | --- | --- | --- | --- | --- | --- | --- | --- | --- |
| 1. | F | 75 | 7.4 | 5 | - | - | No | No | No | No |
| 2. | F | 50 | 12.6 | 3 | - | - | No | No | No | No |
| 3. | M | 19 | 9.2 | 3 | - | Neg | Yes | Yes, 4 days | No | Yes, 10 days |
| 4. | M | 74 | 10.2 | 2 | - | - | No | No | No | No |
| 5. | F | 24 | 10.8 | 2 | - | - | No | No | No | Yes, 20 days |
| 6. | F | 82 | 7.0 | 3 | Pos | Neg | Yes | Yes, 0 days | No | Yes, 74 days |
| 7. | M | 15 | 8.5 | 4 | - | - | No | No | No | Yes, 8 days |
| 8. | F | 5 | 5.3 | 3 | - | Neg | Yes | Yes, 2 days | No | Yes. 2 days |
| 9. | F | 89 | - | 4 | - | - | No | No | No | No |
| 10. | F | 80 | 12.8 | 2 | - | - | No | No | No | No |
| 11. | M | 66 | 13.7 | 3 | - | - | Yes | Yes, 8 days | No | No |
| 12. | M | 53 | 13.4 | 2 | - | - | No | No | No | No |
| 13. | M | 66 | 4.5 | 4 | - | - | No | No | No | No |

*Timing is given with respect to the number of days after study enrolment.

Abbreviations: F=female; M=male; NEG= negative; POS=positive
